# α-Ketoglutarate stimulates cell growth through the improvement of glucose and glutamine metabolism in C2C12 cell culture

**DOI:** 10.3389/fnut.2023.1145236

**Published:** 2023-05-10

**Authors:** Bingquan Yang, Yuefei Liu, Jürgen Michael Steinacker

**Affiliations:** ^1^Department of Endocrinology, Zhongda Hospital, Southeast University, Nanjing, China; ^2^Division of Sports and Rehabilitation Medicine, Department of Cardiology, University of Ulm, Ulm, Germany

**Keywords:** a-ketoglutarate, cell growth, energy metabolism, glucose, glutamin, lactate, ammonia, C2C12

## Abstract

**Introduction:**

Cellular adaptation to physical training and energy metabolism play an important role during physical exercise. This study sought to investigate the effects of α-KG on cell growth and energy metabolism in C2C12 cell culture.

**Methods:**

C2C12 cells were cultured in media pretreated without (control) or with α-KG at different concentrations, and cells and media were harvested every 24 h for 8 days. From cell counts, specific cell growth rate (SGR) and doubling time were calculated. The content of glucose, glutamine, lactate, and ammonia in media was determined, and the specific consumption rate (SCR) or production rate (SPR) was calculated. Additionally, cell colony-forming efficiency (CFE) was determined.

**Results:**

The control cells showed a CFE at 50%, a typical cell growth curve in the first 5 days with a mean SGR at 0.86/day, and a mean cell count doubling time at 19.4 h. In the group with α-KG at 100 mM, the cells underwent rapid cell death, and thus no further analysis was made. The treatment with α-KG at lower concentrations (0.1 mM and 1.0 mM) led to a higher CFE at 68 and 55%, respectively, whereas those in groups with higher α-KG concentration decreased (10 and 6% for 20 mM and 30 mM α-KG, respectively). The mean SGR was 0.95/day, 0.94/day, 0.77/day, 0.71/day, and 0.65/day for groups treated with α-KG at 0.1, 1.0, 10.0, 20.0, and 30.0 mM, respectively, and the corresponding cell count doubling time was 17.6, 17.8, 20.9, 24.6, and 24.7 h, respectively. In comparison with that of the control group, the mean glucose SCR decreased in all the groups treated with α-KG, while the mean glutamine SCR remained unchanged; the mean lactate SPR increased in the groups treated with α-KG ≥ 20.0 mM. Finally, the mean SPR of ammonia was lower in all α-KG groups than that in the control.

**Discussion and conclusion:**

The treatment with α-KG at lower concentrations increased cell growth whereas at higher concentrations decreased cell growth, and α-KG reduced glucose consumption and ammonia production. Therefore, α-KG stimulates cell growth in a dose-dependent manner, which is likely through the improvement of glucose and glutamine metabolism in a C2C12 culture setting.

## 1. Introduction

Energy metabolism plays a determinant role in cellular function and cell biology. In energy metabolism, α-ketoglutarate (α-KG), an intermediate metabolite and analog of amino acid, is involved in the different pathways and has a bridging role (over-talking) between glucose and amino metabolism (1). Studies have shown that α-KG participates in the tri-carbonyl circle as a metabolite and affects the activity of the enzymes that are crucial in energy metabolism (2). Therefore, α-KG has impacts on glucose and amino metabolism as well as lactate and ammonia production (3). Physical training brings about a variety of physiological changes that are involved in energy metabolism, and these changes can, in turn, influence physical performance and training effects (4–6). One of the well-known phenomena is the so-called exercise-induced hyperammonemia which disturbs neurological function, impairs physical performance, and reduces exercise tolerance (7, 8). It is evident that the supplementation of α-KG could improve exercise tolerance and training effects in untrained healthy subjects (9) and help patients with diabetes mellitus perform physical training and control blood glucose (10). However, mechanisms responsible for the potential beneficial effects of α-KG supplement have not yet been thoroughly understood. Trying to understand such mechanisms is of high scientific and practical interest since they might be involved in physical training and doping prevention. Because exercise and training are based on functional and metabolic modifications in terms of cellular adaptation, it is likely that α-KG stimulates cellular processes through effects on energy metabolism. Previous studies have demonstrated that adding α-KG to cell culture media has a profound effect on substrate metabolism (3, 11). In this regard, it is likely that this effect attributes to improved cellular adaptation to physical exercise. We, therefore, conducted the present study to determine the effects of α-KG added to cell culture media on cell growth and glucose as well as amino metabolism in a setting with a C2C12 cell line.

## 2. Materials and methods

### 2.1. Cell culture

#### 2.1.1. Culture medium

Original growth medium (OGM): Dulbecco's Modified Eagle Medium (DMEM, Lot No. 722027, Gibco Invitrogen Company, Berlin, Germany) with 10% heat-inactivated fetal bovine serum, 1% penicillin, and 1% streptomycin. The components of DMEM are presented in Table 1.

**Table 1:** Components of the original growth media.

**Components**	**Molecular weight**	**Concentration (mg/L)**	**Concentration (mM)**
Glycine	75	30	0.4
L-Arginine hydrochloride	211	84	0.398
L-Cystine 2HCl	313	63	0.201
L-Glutamine	146	580	3.97
L-Histidine hydrochloride-H_2_O	210	42	0.2
L-Isoleucine	131	105	0.802
L-Leucine	131	105	0.802
L-Lysine hydrochloride	183	146	0.798
L-Methionine	149	30	0.201
L-Phenylalanine	165	66	0.4
L-Serine	105	42	0.4
L-Threonine	119	95	0.798
L-Tryptophan	204	16	0.0784
L-Tyrosine	181	72	0.398
L-Valine	117	94	0.803
Choline chloride	140	4	0.0286
D-Calcium pantothenate	477	4	0.00839
Folic acid	441	4	0.00907
Niacinamide	122	4	0.0328
Pyridoxine hydrochloride	204	4	0.0196
Riboflavin	376	0.4	0.00106
Thiamine hydrochloride	337	4	0.0119
i-Inositol	180	7.2	0.04
Calcium chloride	147	264	1.8
Ferric nitrate	404	0.1	0.000248
Magnesium sulfate	246	200	0.813
Potassium chloride	75	400	5.33
Sodium bicarbonate	84	3,700	44.05
Sodium chloride	58	6,400	110.34
Sodium phosphate monobasic	154	141	0.916
D-Glucose	180	4,500	25
Phenol red	376.4	15	0.0399
Sodium pyruvate	110	110	1

Intervention growth medium (IGM): The different amounts of α-KG salts (generously provided by Evonik Industries^®^, Essen, Germany) were dissolved in OGM to attain the final concentration of α-KG at 0.1 mM, 1 mM, 10 mM, 20 mM, 30 mM, and 100 mM (Table 2). The pH value of the IGM was adjusted with 1 mM sodium hydroxide to match that of the OGM. All the media were sub-packed into sterile 50 ml polystyrene conical tubes (Falcon, Becton Dickinson France S.A.S., Le Pont-de-Claix Cedex, France) and were deposited in a 4°C refrigerator.

**Table 2:** α-ketoglutarate concentration and pH.

**Group**	**α-KG final concentration (mM)**	**pH**
A (Control)	0	7.79
B	0.1	7.75
C	1	7.68
D	10	7.11
E	20	6.53
F	30	5.99
G	100	4.36

#### 2.1.2. Cell line and primary cell culture process

The C2C12 cell line was purchased from the LGC Standards GmbH (Wesel, Germany). The vials of frozen C2C12 cells (1 × 10^6^) were thawed in a 37°C water bath with constant agitation (1 min). Then, the outside of the vials was wiped with 70% ethanol. The contents of the vials were transferred to prepared culture plates (150 mm × 150 mm, Nunc OmniTray) with 15 ml OGM and then incubated at 37°C and 8% CO_2_ humidified (Heinicke incubator Model 7341, SPW Industrial, California, USA). The cell cultivation was observed using a microscope daily. Culture media were changed 1 day after seeding and every 2 days thereafter until the cells reached ~70% confluence. Before sub-culture, the cells were washed three times with cold Dulbecco's phosphate-buffered saline (DPBS, Lot No. H00210-0211, Gibco Invitrogen Company, Massachusetts, USA) and then treated using 0.25% trypsin solution (Lot No. 9D0702, Sigma Company, St. Louis, Missouri, USA) containing 0.01% ethylenediaminetetraacetic acid. After re-suspension with the culture medium, viable cell counting was performed with trypan blue exclusion (see below). By reaching sufficient counts for the experimental protocol, the cells were distributed (see below). Stock cultures of the cells remained in OGM.

### 2.2. Study design

**Figure 1:**
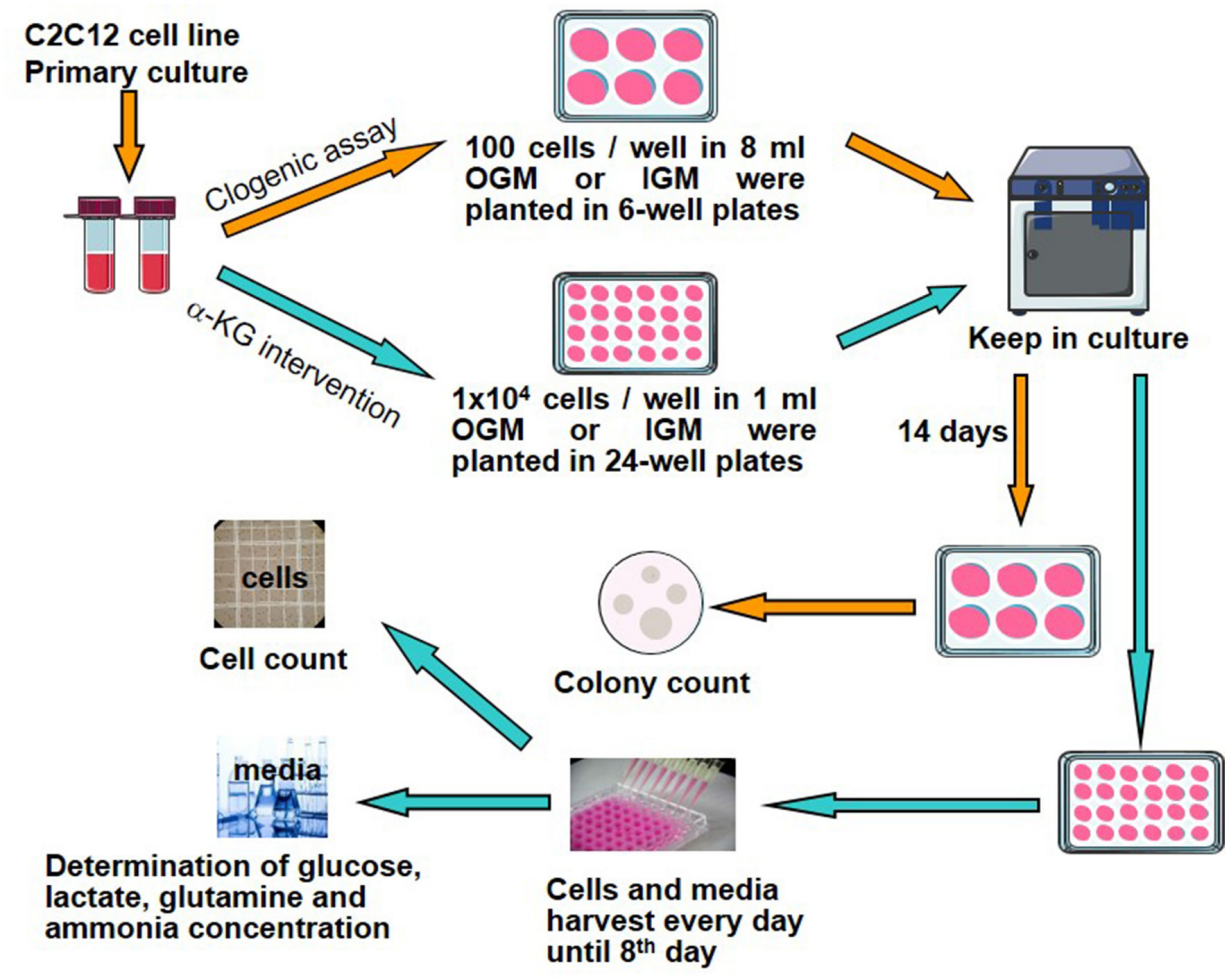
Study design and experimental protocol. α-KG, α-ketoglutarate; OGM, original growth media; IGM, intervention growth media.

To evaluate the effects of α-KG on C2C12 cell growth and their relation to metabolites including glucose, glutamine, lactate, and ammonia involved in energy metabolisms, clonogenic assays, cell counting, and collection of cell culture media were performed according to the following procedure (Figure 1).

### 2.3. Clonogenic assay

C2C12 cells of the exponential growth phase during the primary cell culture were harvested and then treated using trypsinization. After adding the medium, the cells were collected and detached to produce a single-cell suspension. Then, the cells were washed with DPBS several times to get rid of the rudimentary trypsin solution. Cell counting was undertaken, and the cell suspension was diluted with OGM to a concentration of 1 × 10^4^/ml. In total, 10 μl cell suspension (100 cells) was pipetted into each well of the 6-well culture plates (35 mm × 35 mm each), according to the protocol of the clonogenic assay (12) (Figure 1). There were a total of 21 wells seeded with C2C12 cells. Another 5ml OGM was supplemented to each culture well. These plates were put back into the incubator at 37°C and 8% CO_2_. When these cells were attached to the bottom of culture plates, suspensions were discarded, and another 8 ml OGM or different kinds of IGM were replaced to each culture well correspondingly. After the media were replaced, these cells were put back into the incubator again. Then, the cells were checked under the microscope daily until sufficient colony formation at the end of the 14th day.

At the end of the 14th day, the culture media were discarded, and the cells were rinsed carefully with DPBS. The plates containing the cells were given by 3 ml mixture solution of 6.0% glutaraldehyde and 0.5% crystal violet to fix and stain cell colonies for 30 min. The plates were rinsed with water and dried at room temperature.

Colonies were enumerated and characterized according to their morphology under an inverted microscope and a 100 mm transparent paper marked with a scoring grid. A colony was considered as formed when ≥ 50 cells were found. Colony-forming efficiency (CFE) was calculated using the following formula:


$$\begin{array}{c} CFE=\left(numberofcoloniesformed/numberofcellsseeded\right)\times \;100\%. \end{array}$$

A total of seven groups were found (Table 2). Every group had three wells and was duplicated. Thus, altogether, there were six cell colony samples obtained in each group at the end of the experiments.

### 2.4. Cell counting and cell growth calculation

The cells of the exponential growth phase during the primary culture were taken for further cell culture, and the cell suspension was diluted with OGM to attain 1 × 10^6^ cells/ml. In total, 10 μl cell suspension (1 × 10^4^ cells) was pipetted into each well of 24-well culture plates (16 mm × 16 mm each), according to the intervention experimental design (Figure 1). There were a total of seven culture plates (a total of 168 wells). In addition, 0.99 ml of OGM or IGM at assigned α-KG concentrations (Table 2) was added to each culture well.

The viable cells were counted using trypan blue exclusion methods (13). After rinsed with DPBS, the cells of each well were trypsinized with 1 ml of trypsin solution (5% trypsin with 2% EDTA, Lot No. 9D0702, Sigma Company, St. Louis, Missouri, United States). After re-suspension with trypsin solution, the cells were collected into a 10 ml centrifuge tube and then rinsed with DPBS two times. The mixed solution was centrifuged at 1,000 rpm for 10 min at room temperature. The supernatant was discarded, and the cell pellet was re-suspended thoroughly with 1 ml of DPBS. In total, 45 μl of cell suspension and 5 μl of trypan blue (0.4%, Lot No. 076K2331, Sigma Company, St. Louis, Missouri, USA) were mixed and incubated for 4 min at room temperature. After incubation, a drop of the stain/culture combination was added on both sides of the hemocytometer with a cover slip (0.0025 mm^2^/grid, Lab Logistics Group, Meckenheim, Germany). Total viable cells (opaque) were counted in four 1 mm × 1 mm hemocytometers under an invert microscope (Axiovert 25 CFL, Carl Zeiss Company, Oberkochen, Germany) and calculated as follows:

Total viable cell numbers/ml = total opaque cell counts in four squares × 2.5 × 10^3^ × dilutions = total opaque cell counts in four squares × 2.5 × 10^3^ × (45 + 5) / 45 = 2.778 × 10^3^ × total opaque cell counts in four squares.

Based on cell counts, a cell growth curve over a time course can be depicted and is useful in evaluating the growth characteristics of a cell line. Lag time and population doubling time (t_d_) can be determined by a specific growth curve (14). From the cell growth curve, the cell-specific growth rate (SGR) can be calculated as follows (15):


$$\begin{array}{c} \mathrm{SGR}=ln\left(C_{\text{t}2}/C_{\text{t}1}\right)/\left(t2-t1\right) \end{array}$$

where C_t1_ and C_t2_ are cell counts at the points t1 and t2, respectively, and t2-t1 is the time interval from t1 to t2.

For a constant cell growth rate, the formula for the doubling time of cell counts t_d_ is given as follows:


$$\begin{array}{c} t_{\text{d}}=ln\left(2\right)/SGR=\;0.693/SGR. \end{array}$$

From our previous study (16), we observed that the cell count followed a curve with an increasing phase from culture day 1 to culture day 5 and then with a decreasing phase thereafter. Therefore, we focused on cell count data during the cell growth phase (culture days 1 to 5).

### 2.5. Determination of metabolites

The metabolites determined were glucose, lactate, glutamine, and ammonia.

The lactate and glucose concentrations of the medium samples were determined using the enzymatic-amperometric method (17, 18). A total of 20 μl of each sample was added to the tube containing 0.5 ml reagent solution. After a thorough mixture, the tubes were placed in the diagnostic instrument (BIOSEN S line, EKF-diagnostic, Barleben, Germany) for automatic measurement, and the concentration of lactate and glucose was simultaneously reported. The measurement was reported in duplicate.

The specific consumption rate (SCR) of glucose and the specific production rate (SPR) of lactate were then calculated by simple formulas as follows (Ozturk and Palsson 1990):


$$\begin{array}{c} -d\left[Glc\right]/d\text{t}={\text{k}}_{\text{Glc}}\;{\text{X}}_{\text{v}}\\ d\left[Lac\right]/d\text{t}={\text{k}}_{\text{Lac}}\;{\text{X}}_{\text{v}} \end{array}$$

where d[Glc] and d[Lac] are the differences in glucose and lactate concentrations between the assigned two time points; *k*_Glc_ and *k*_Lac_ are the constants of glucose consumption and lactate production, respectively, and calculated by the differential method of (19); *X*_v_ is the viable cell counts, and *t* is time (we took 5 days of the exponential cell growth phase).

The determination of glutamine and ammonia concentration was based on enzymatic analysis (20). We used the L-glutamine/ammonia assay kit (Lot No. 100809-1, Megazyme Company, Wicklow, Ireland), and the reagents were prepared according to the protocol of the assay kit. Standard ammonia and glutamine solutions were prepared as the quality control solutions.

The absorbance was read at 340 nm wavelength using the spectrophotometer (Ultrospec III, Pharmacie Company, Hamburg, Germany).

Similar to that for glucose consumption and lactate production, the specific rate of glutamine consumption (d[Gln] / d*t*) and ammonia production (d[Ammo] / d*t*) was calculated, respectively.

### 2.6. Statistical analysis

The raw data of the repeated measurements were derived from the mean values. There were six raw data (three wells with two experiments) in each group analyzed. Statistical analysis was performed with SPSS version 17.0 (SPSS Inc., Chicago, IL, USA). The results of statistical analysis are expressed as mean ± standard deviation (SD). The univariate analysis of variance (ANOVA) process of the generalized linear model (GLM) was chosen for the data to compare the integrated differences among the groups. The profile plots of estimated marginal means of the measured data were made by the GLM. Comparisons between different groups at the same levels of time factor or different time points within the same group, the least-significant difference (LSD), and Student–Newman–Keuls (S-N-K) of the one-way ANOVA test were adopted for the data with normal distribution; otherwise, the non-parametric statistics were performed. A statistical significance was considered at a *p-value* of < 0.05.

## 3. Results

### 3.1. Cell colony-forming efficiency

**Figure 2:**
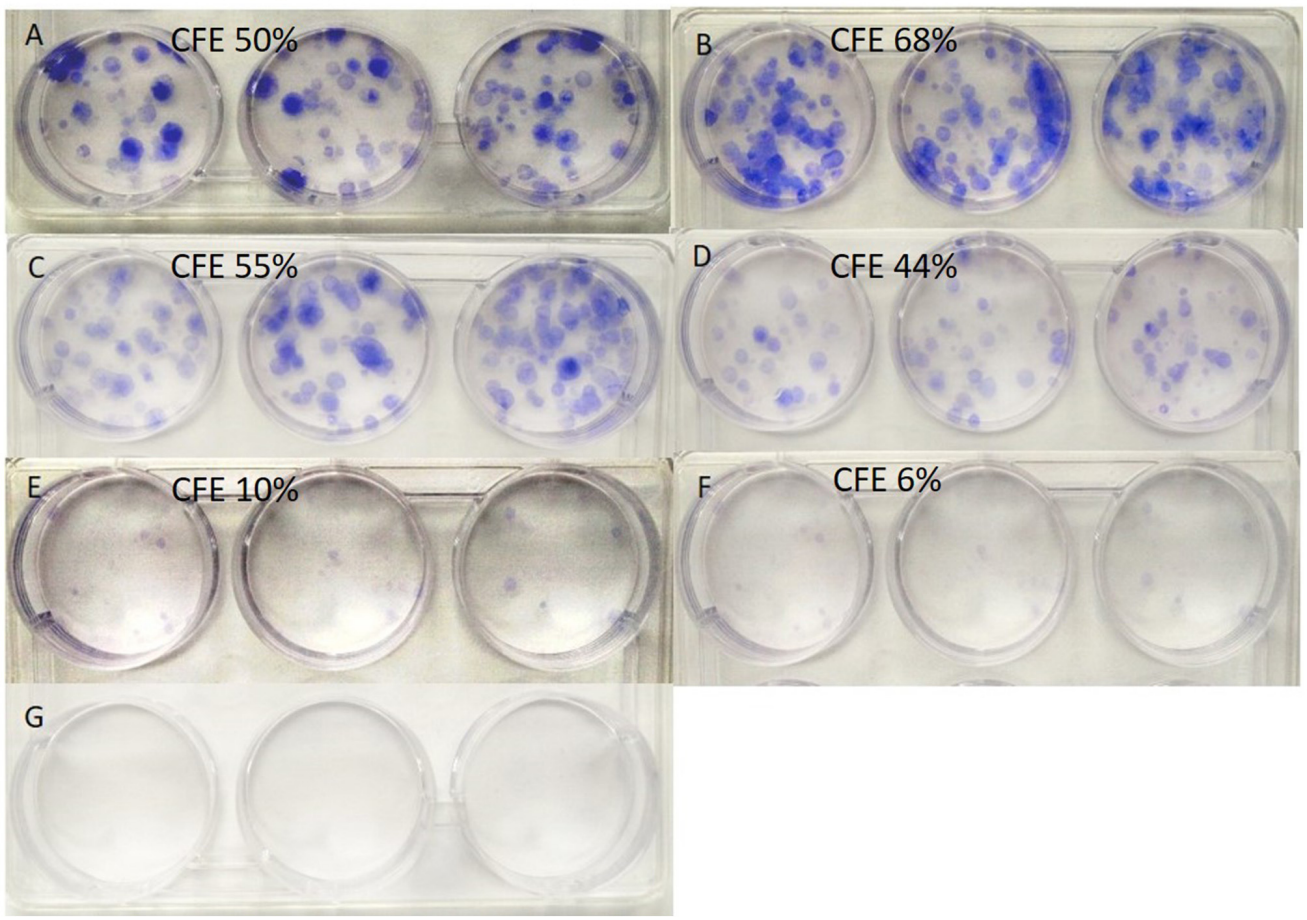
Colonies formed by cells growing from single-seeded cells. α-KG, α-ketoglutarate. **(A–G)** indicate the groups (see Table 2), and the calculated colony-forming efficiency (CFE), respectively.

The cell colonies formed during the culture process from a single-seeded cell are presented in Figure 2. The calculated colony-forming efficiency was 50, 68, 55, 44, 10, and 6% for groups A to F, respectively. While the colony-forming efficiency in groups B and C (treated with α-KG at 0.1 mM or 1 mM) was higher than that of the control, the treatment with higher α-KG (≥10 mM) resulted in a decreased colony-forming efficiency.

### 3.2. Cell growth analyses

**Figure 3:**
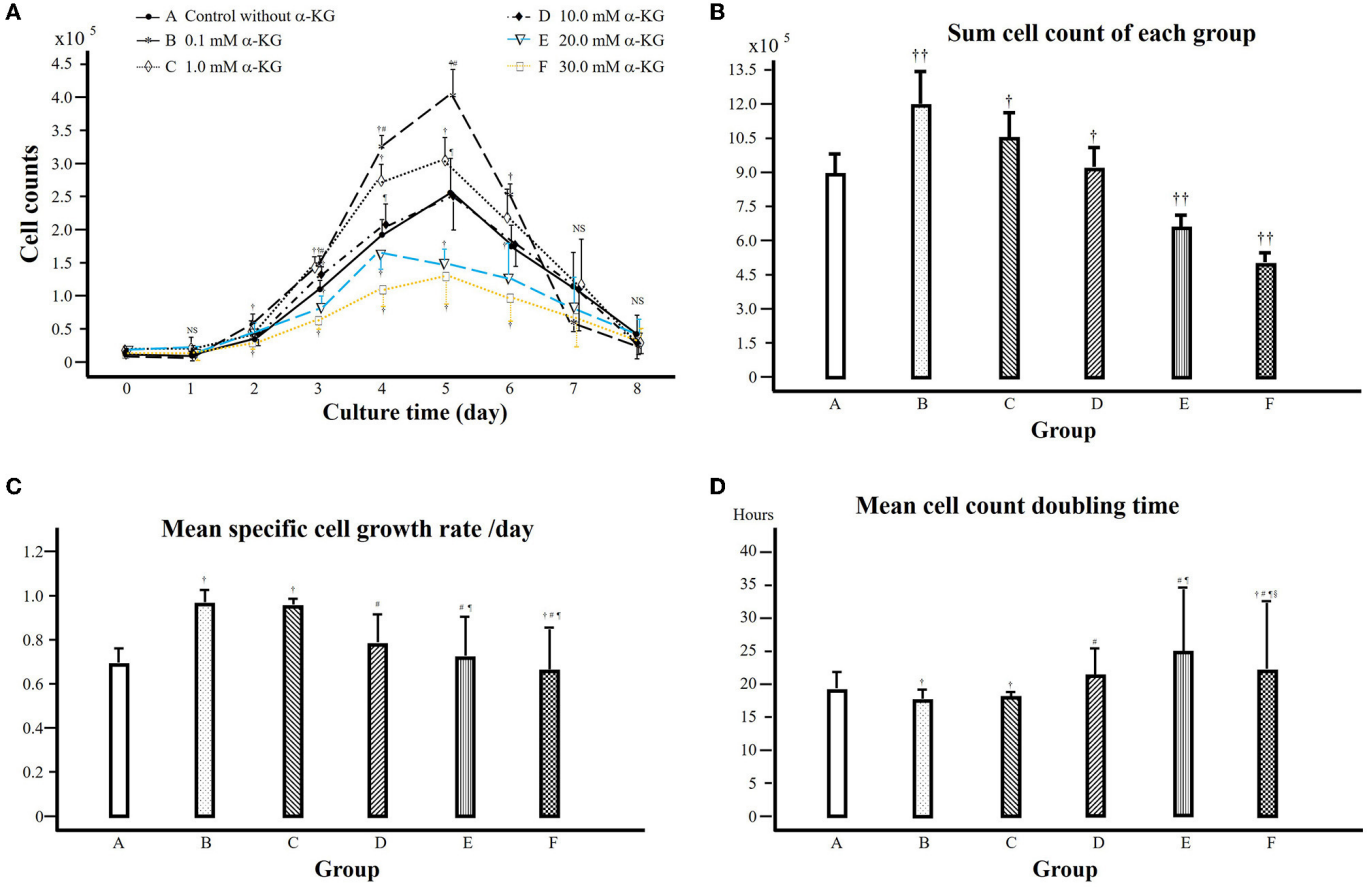
**(A)** Cell counts from groups A–F treated with α-KG at different concentrations (A, control; B–F, treated with α-ketoglutarate). 10^5^ cells were seeded on day 0 for culture. The cultured cells were harvested every 24 h, i.e., days 1–8, and counted under a microscope. † in comparison with that of control at the same time point, *P* < 0.05; # in comparison with that of group B, *P* < 0.05; ¶ in comparison with that of group C, *P* < 0.05. **(B)** Sum cell count of each group. † in comparison with group A, *P* < 0.05; ††: in comparison with group A, *P* < 0.01. **(C)** Mean specific cell growth rate over 5 days during the exponential cell growth phase. † in comparison with that of the control at the same time point, *P* < 0.05; # in comparison with that of group B, *P* < 0.05; ¶ in comparison with that of group C, *P* < 0.05. **(D)** Cell count doubling time during the exponential growth phase. † in comparison with that of the control at the same time point, *P* < 0.05; # in comparison with that of group B, *P* < 0.05; in comparison with that of group C, *P* < 0.05; § in comparison with that of group D, *P* < 0.05.

In the cell culture, a typical cell growth curve during the growth phase in cultivation could be observed in the control group (Figure 3A), where the cell counts increased from the 2nd culture day, reached their peak on the 5th culture day, and then decreased afterward (Supplementary Table 1). Compared to that of the control group, the cell counts increased significantly faster and obtained a higher level in groups B and C, while those of groups E and F showed slower growth and obtained depressed cell count levels. A similar curve of the cell culture to control could be obtained from group D.

We have analyzed the sum cell counts for each group during the cell growth phase (culture days 1 to 5) (Figure 3B). It is shown that in comparison with that of the control group, the sum cell counts in groups B and C were significantly higher, while the sum cell counts in groups E and F were clearly lower.

From cell counts data, we have further analyzed the specific cell growth rate for the growth phase during cell culture (days 1 to 5) (Figure 3C). Compared to that of the control, the specific cell growth rate was higher in groups B and C but lower in group F (*P* < 0.05). The further comparison among the groups treated with α-KG shows that the treatment with higher α-KG (at ≥10 mM) decreased the specific cell growth rate (compared to those of lower α-KG treatment—groups B and C).

The results of cell growth rates can be further reciprocally represented by the doubling time of cell counts (Figure 3D), where the highest level of the specific growth rate in group B is reciprocally represented by the shortest doubling time of cell counts (*P* < 0.05).

### 3.3. Glucose and glutamine consumption

**Figure 4:**
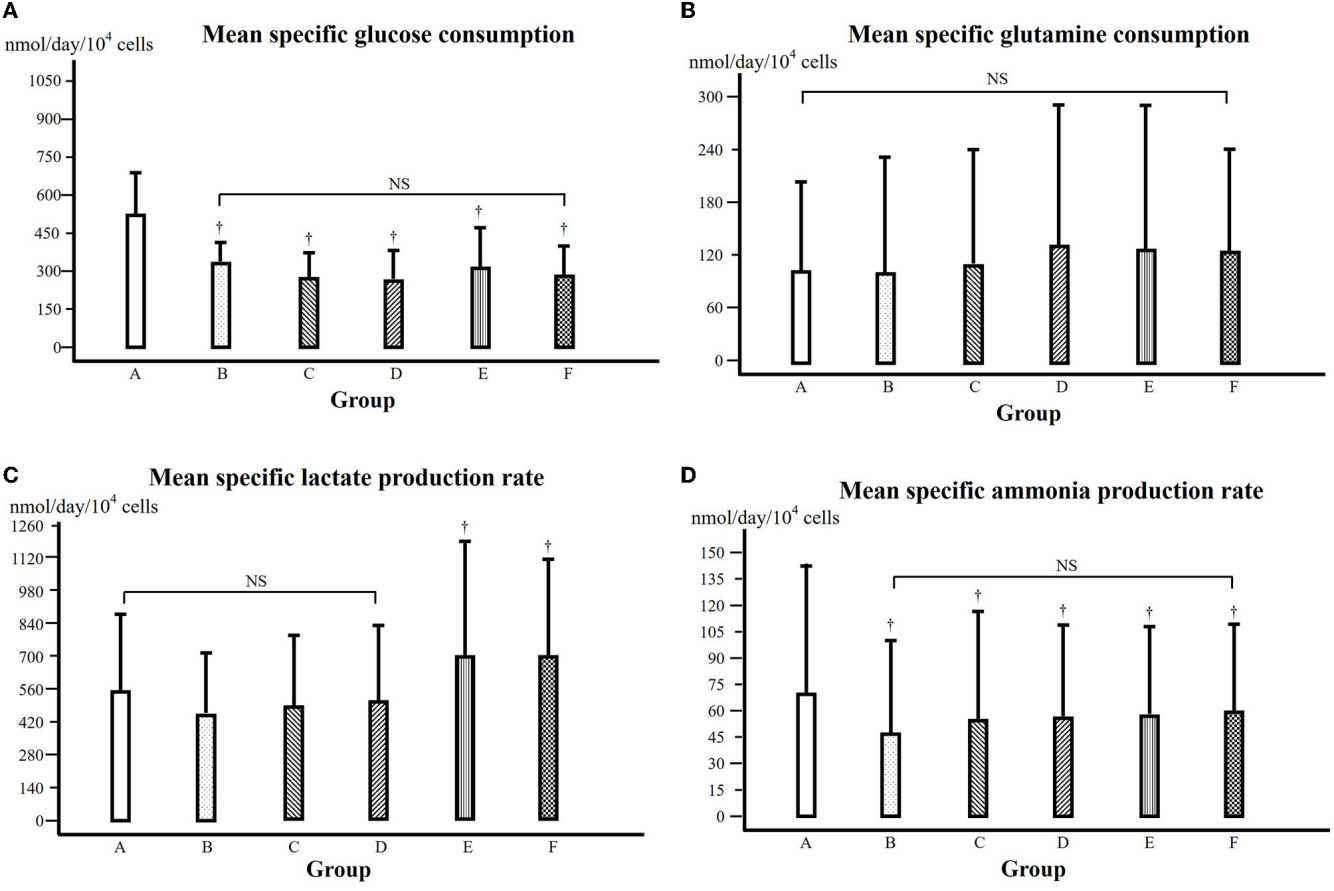
**(A)** Mean specific glucose consumption over 5 days during the exponential cell growth phase (days 1 to 5). † in comparison with that of the control at the same time point, *P* < 0.05. NS: comparison among the groups, not significant. Groups A–F were treated with α-KG at different concentrations (A, control; B–F, treated with α-ketoglutarate). **(B)** Mean specific glutamine consumption over 5 days during the exponential cell growth phase (days 1 to 5). NS: comparison among the groups, not significant. **(C)** Mean specific lactate production over 5 days during the exponential cell growth phase (days 1 to 5). † in comparison with that of groups A, B, C, and D, respectively, at the same time point, *P* < 0.05. NS: comparison among the groups, not significant. **(D)** Mean specific ammonia production over 5 days during the exponential cell growth phase (days 1 to 5). † in comparison with that of the control at the same time point, *P* < 0.05. NS: comparison among the groups, not significant.

From the data of glucose content of cell culture media (see Supplementary Table 2), the specific glucose consumption of each day during the cell growth phase (culture days 1 to 5) (see Supplementary Table 3), from which the mean specific growth rate from the 5 days of cell growth phase could be calculated and is shown in Figure 4A. Compared to that of the control, the specific glucose consumption was significantly lower in all the groups treated with α-KG (*P* < 0.05).

From the data on the glutamine content of cell culture media (see Supplementary Table 4), the specific glutamine consumption of each day during the cell growth phase (culture days 1 to 5) could be calculated (see Supplementary Table 5). Then, the mean specific glutamine consumption could be calculated and is shown in Figure 4B, where there was no statistically significant difference among groups A, B, and C but higher in groups D, E, and F (*P* < 0.05).

### 3.4. Lactate and ammonia production

The lactate content of the cell culture media is presented in Supplementary Table 6. From these data, the specific lactate production of each day during the cell growth phase could be obtained (see Supplementary Table 7), from which the mean specific lactate production rate could be calculated and is shown in Figure 4C. In comparison with that of the control, the mean specific lactate production of groups B, C, and D was somewhat but not statistically significantly lower; however, the mean specific lactate production of groups E and F was significantly higher (*P* < 0.05).

The ammonia content of the cell culture media is presented in Supplementary Table 8. We further calculated the specific ammonia production during the cell growth phase (see Supplementary Table 9), and the mean specific ammonia production is shown in Figure 4D. In comparison with that of the control, the mean specific ammonia production was lower in all groups treated with α-KG, with the lowest in group B (*P* < 0.05).

## 4. Discussion

Physical exercise brings about a variety of physiological challenges that are critically involved in metabolism (21, 22). Nutrients are essential for energy metabolism, and supplementation of nutrients has been proven to significantly influence exercise performance and training effects (23–25). One of our previous studies has shown that the supplementation of α-KG can improve exercise tolerance and thereby augment the training effects (9). A further study shows that through the supplementation of α-KG, patients with type 2 diabetes mellitus could get better blood glucose control through improved physical training, where this effect was possibly attributed to a modulation of ammonia metabolism (10). However, up to date, there is a lack of studies dealing with mechanisms responsible for the supportive effect of α-KG supplementation on physical training in this aspect.

Undoubtedly, the training effect is based on cellular adaptation and energy metabolism. We have, therefore, conducted this study to explore possible mechanisms contributing to the beneficial effect of physical training through α-KG supplementation.

The clonogenic assay is an *in vitro* cell survival assay based on the ability of a single cell to grow into a colony (26). From this morphological experiment (Figure 2), we observed that the formation rates of cell colonies originating from single-seeded cells were obviously different among the groups. In comparison with that of the control group (50%), the calculated colony-forming efficiency was higher in the groups treated with α-KG at 0.1 (68%) and 1.0 mM (55%), respectively, whereas that in groups treated with higher concentrations of α-KG (i.e., ≥10.0 mM, the colony-forming rates ≤ 44%) was lower. These data suggest that the colony-forming efficiency was affected by the treatment of α-KG, where the effects varied in a dose-dependent manner.

Furthermore, we have conducted quantitative analyses on cell growth curves based on cell count during the cell culture procedure. Figure 3 shows that during cell culture, the cell growth underwent an exponential growth phase (cell growth phase, culture days 1 to 5) at first, and from the cell counts, we obtained the total cell counts of each group (Figure 3B). In comparison with that of the control, the sum cell count of the group treated with α-KG at 0.1 or 1.0 mM was significantly higher (P < 0.05), whereas that of the group treated with α-KG at 20 mM or 30 mM was lower (*P* < 0.01). The calculated-specific cell growth rate shows similar results (Figure 3C), which is further reflected reciprocally by the doubling time of the cell counts (Figure 3D). All these results demonstrate that the treatment with α-KG brought about distinct effects on cell growth, i.e., stimulating cell growth at a relatively lower concentration but impairing cell growth when α-KG concentration was higher, indicating a clear dose-dependent effect on the cell growth.

Our results mentioned above seems to be consistent with the previous study reported by Hassell and Butler, where α-KG at 4 mM in the medium could increase cell yield by 17% in maximum cell counts (3). Mailloux et al. found that supplementing 0.5 to 5 mM α-KG in aluminum-exposed hepatocytes cultural medium could improve the hepatocyte viability significantly (2). To the best of our knowledge, to date, there is no study on α-KG treatment at higher than 10 mM concentration was reported. In the present study, we observed that the treatment of α-KG at higher concentration (≥ 20 mM) led to impaired cell growth (Figures 2, 3). A depressed pH value through α-KG addition to the media (Table 2) is not responsible for this result because the pH value of the intervention media was adjusted accordingly. Whether a possible influence is due to the potentially changed osmolality of the culture media or whether there is a potential toxic effect of α-KG at too high a concentration remains unexplained, which certainly needs further investigation. Altogether, we can conclude that the treatment with α-KG at a lower concentration (≤ 10 mM) is suitable to stimulate cell growth in a C2C12 cell culture.

Cell growth is certainly based on energy metabolism. During the cell culture procedure, different substrates or nutrients are used up, and the major energy source for the C2C12 cell growth is glucose and glutamine (27), and therefore, we have determined the contents of the culture media and further calculated their consumption (Figures 4A, B). It is shown in Figure 4A that the specific glucose consumption, i.e., glucose consumption for a certain cell amount and culture time, was significantly lower in all the groups treated with α-KG (*P* < 0.05). This means that for growing a sufficient amount of cells, less glucose was consumed in the presence of additional α-KG. This result suggests that α-KG treatment significantly improved the efficacy of glucose consumption during cell growth. This is consistent with the previous study reported by Frame and Hu (28).

Considering that glucose consumption is one of the major energy sources for cell growth, an improved efficacy of glucose consumption would mean that the glucose was more efficiently oxidized, or in other words, less lactate would be produced. We observed that the specific lactate production in the groups treated with α-KG at lower concentration (Figure 4C), indicating therefore that the improved efficacy of glucose utilization went through the oxidative pathway. Of course, caution has to be taken since the lowered lactate production in groups treated at lower α-KG concentration was statistically not significant compared to that of the control.

Glutamine consumption is thought of as the other energy source in this experimental setting. However, we found no significant difference for the groups treated with α-KG at lower concentrations but higher for the groups treated with α-KG at higher concentrations in comparison with that of the control (Figure 4B). Furthermore, the specific ammonia production was significantly depressed in the groups treated with α-KG (*P* < 0.05, Figure 4D). This might imply that glutamine was mainly used for constituting cell structure rather than providing energy in this study setting. It is already known that through transamination, α-KG can bind ammonia to form glutamate, and in this way, α-KG can affect ammonia production through its scavenger role. A lowered specific glutamine consumption and ammonia production suggest that less glutamine was involved in the energy metabolism, and this must be due to improved efficacy of glucose utilization through α-KG supplementation.

## Data availability statement

The original contributions presented in the study are included in the article/Supplementary material, further inquiries can be directed to the corresponding author.

## Author contributions

BY designed the study, performed the experiment, and carried out data analyses. YL designed the study and conducted partial data analyses. JS took supervision and administrative tasks. All the authors contributed to the composition of the article and approved the submitted manuscript.
