# Supplementary material for: α-Ketoglutarate stimulates cell growth through the improvement of glucose and glutamine metabolism in C2C12 cell culture
Source: Front Nutr. 2023 May 10;10:1145236. doi: 10.3389/fnut.2023.1145236 (PMC10208397; doi:10.3389/fnut.2023.1145236)
Supplement: Supplementary file 1 [file Table_1.DOCX]

| Group | Initially  Seeded | Day 1 | Day 2 | Day 3 | Day 4 | Day 5 | Day6 | Day7 | Day8 |
| --- | --- | --- | --- | --- | --- | --- | --- | --- | --- |
| A | 10000 | 8800±1556 | 34003±2188 | 107954±8612 | 190641±18091 | 250580±46242 | 167420±26926 | 110550±45620 | 41360±23190 |
| B | 10000 | 10120±2508 | 46420±4375^∆^ | 146542±5939^∆,^ | 312070±11619^∆^ | 388850±35444^∆^ | 243760±12556^∆,^ | 54670±7103 | 21230±10577 |
| C | 10000 | 11440±984 | 40348±2311 | 136444±6719^∆,¶^ | 262570±16712^∆,¶^ | 300080±27449^∆,¶^ | 203060±37055 | 102960±61357 | 17050±8783 |
| D | 10000 | 9240±3615 | 41580±9443 | 128964±4768^∆,¶^ | 203830±25179^¶,‡^ | 243540±42021^¶,‡^ | 178200±28001^¶^ | 100760±58624 | 29700±19289 |
| E | 10000 | 8360±1840 | 39688±11311 | 78144±9590^∆,¶,‡,†^ | 159060±16593^∆,¶,‡,†^ | 146520±15891^∆,¶,‡,†^ | 126500±46500^¶,‡,†^ | 77770±37504 | 33880±19718 |
| F | 10000 | 8360±3935 | 28358±2252^¶,‡,†,§^ | 56892±6699^∆,¶,‡,†,§^ | 103950±10436^∆,¶,‡,†,§^ | 128260±31438^∆,¶,‡,†^ | 94380±27083^∆,¶,‡,†^ | 62150±34763 | 24400±14538 |
