# Supplementary material for: α-Ketoglutarate stimulates cell growth through the improvement of glucose and glutamine metabolism in C2C12 cell culture
Source: Front Nutr. 2023 May 10;10:1145236. doi: 10.3389/fnut.2023.1145236 (PMC10208397; doi:10.3389/fnut.2023.1145236)
Supplement: Supplementary file 2 [file Table_2.DOCX]

| Group | Baseline | Day 1 | Day 2 | Day 3 | Day 4 | Day 5 | Day6 | Day7 | Day8 |
| --- | --- | --- | --- | --- | --- | --- | --- | --- | --- |
| A | 23.02±0.52 | 22.35±.063 | 21.15±0.56 | 18.86±0.66 | 13.87±1.03 | 10.95±0.19 | 8.78±0.68 | 9.19±1.81 | 9.16±0.36 |
| B | 22.80±0.67 | 22.28±0.69 | 21.22±0.65 | 19.02±1.10 | 13.40±1.44 | 10.34±0.60 | 7.88±0.95 | 9.40±0.49 | 8.34±1.14 |
| C | 22.86±0.71 | 22.38±0.84 | 21.27±1.17 | 19.35±1.63 | 15.23±3.16 | 12.91±3.46^¶^ | 10.16±2.35 | 11.46±2.78 | 10.94±2.37^∆,¶^ |
| D | 22.89±0.74 | 22.36±0.84 | 21.57±0.80 | 20.17±1.59 | 16.81±2.55^∆,¶^ | 15.17±2.48^∆,¶,‡^ | 13.00±1.52^∆,¶,‡^ | 14.57±1.20^∆,¶,‡^ | 12.15±1.12^∆,¶^ |
| E | 22.99±0.74 | 22.67±0.78 | 21.40±0.98 | 20.02±1.07 | 18.14±1.18^∆,¶,‡^ | 17.51±1.23^∆,¶,‡^ | 17.04±2.25^∆,¶,‡,†^ | 17.49±2.49^∆,¶,‡,†^ | 17.74±0.98^∆,¶,‡,†^ |
| F | 23.19±0.51 | 22.94±0.54 | 22.57±0.61^∆,¶,‡,§^ | 20.48±1.36 | 18.69±1.16^∆,¶,‡^ | 18.32±1.16^∆,¶,‡,†^ | 18.63±2.86^∆,¶,‡,†^ | 20.80±1.15^∆,¶,‡,†,§^ | 19.91±1.29^∆,¶,‡,†,§^ |
