# Supplementary material for: α-Ketoglutarate stimulates cell growth through the improvement of glucose and glutamine metabolism in C2C12 cell culture
Source: Front Nutr. 2023 May 10;10:1145236. doi: 10.3389/fnut.2023.1145236 (PMC10208397; doi:10.3389/fnut.2023.1145236)
Supplement: Supplementary file 3 [file Table_3.DOCX]

| Group | Day 1 | Day 2 | Day 3 | Day 4 | Day 5 |
| --- | --- | --- | --- | --- | --- |
| A | 397.57±176.38 | 568.80±147.15 | 278.76±36.63 | 196.97±13.15 | 177.59±22.96 |
| B | 287.92±136.54 | 299.75±196.98 | 211.03±20.56 | 151.62±20.48^∆^ | 139.47±27.95 |
| C | 200.16±54.93^∆^ | 354.04±335.22 | 176.46±71.50^∆^ | 121.79±19.32^∆^ | 115.50±33.26^∆^ |
| D | 174.92±89.04^∆^ | 382.82±256.86 | 170.02±62.56^∆^ | 114.53±26.67^∆^ | 106.14±27.26^∆^ |
| E | 243.97±128.05 | 476.85±295.89 | 211.17±111.40 | 106.16±35.64^∆,¶^ | 89.52±22.15^∆^ |
| F | 212.47±192.86^∆^ | 427.97±187.74 | 222.43±66.76 | 140.97±30.61^∆^ | 156.03±83.43^§^ |
