# Supplementary material for: α-Ketoglutarate stimulates cell growth through the improvement of glucose and glutamine metabolism in C2C12 cell culture
Source: Front Nutr. 2023 May 10;10:1145236. doi: 10.3389/fnut.2023.1145236 (PMC10208397; doi:10.3389/fnut.2023.1145236)
Supplement: Supplementary file 4 [file Table_4.DOCX]

| Group | Baseline | Day 1 | Day 2 | Day 3 | Day 4 | Day 5 | Day6 | Day7 | Day8 |
| --- | --- | --- | --- | --- | --- | --- | --- | --- | --- |
| A | 2.61±0.33 | 2.37±0.35 | 1.81±0.23 | 1.52±0.18 | 1.06±0.09 | 0.86±0.08 | 0.75±0.12 | 0.84±0.50 | 0.70±0.29 |
| B | 2.59±0.33 | 2.16±0.37 | 1.83±0.28 | 1.61±0.20 | 1.28±0.22^∆^ | 0.93±0.05 | 0.70±0.25 | 1.10±0.45 | 1.28±0.93 |
| C | 2.73±0.53 | 2.25±0.31 | 1.80±0.20 | 1.54±0.08 | 1.24±0.18 | 0.95±0.13 | 1.01±0.41 | 0.90±0.38 | 0.72±0.12 |
| D | 2.71±0.47 | 2.09±0.06 | 1.85±0.06 | 1.45±0.17 | 1.10±0.07 | 0.95±0.06 | 1.07±0.37 | 1.00±0.21 | 1.17±0.37 |
| E | 2.58±0.49 | 2.16±0.25 | 1.82±0.12 | 1.62±0.08 | 1.43±0.08^∆,†^ | 1.17±0.11^∆,¶,‡,†^ | 1.20±0.15^¶^ | 1.50±0.50^∆,‡^ | 1.49±0.55^∆,‡^ |
| F | 2.79±0.48 | 2.44±0.29 | 2.13±0.25^∆,¶,‡,†,§^ | 1.78±0.27^∆,‡,†^ | 1.55±00.24^∆,¶,‡,†^ | 1.28±0.27^∆,¶,‡,†^ | 1.48±0.59^∆,¶,‡^ | 1.57±0.76^∆,‡^ | 1.46±0.45^∆,‡^ |
