# Supplementary material for: α-Ketoglutarate stimulates cell growth through the improvement of glucose and glutamine metabolism in C2C12 cell culture
Source: Front Nutr. 2023 May 10;10:1145236. doi: 10.3389/fnut.2023.1145236 (PMC10208397; doi:10.3389/fnut.2023.1145236)
Supplement: Supplementary file 5 [file Table_5.DOCX]

| Group | Day 1 | Day 2 | Day 3 | Day 4 | Day 5 |
| --- | --- | --- | --- | --- | --- |
| A | 212.16±53.39 | 210.42±69.41 | 52.26±14.27 | 24.04±5.87 | 15.51±4.66 |
| B | 315.54±93.33 | 134.13±25.27 | 31.21±7.79 | 13.62±3.63^∆^ | 8.71±2.75^∆^ |
| C | 301.37±226.93 | 182.70±89.64 | 38.52±14.21 | 15.80±4.68 | 9.48±3.16^∆^ |
| D | 379.01±258.41 | 204.15±102.47 | 45.75±13.08 | 18.59±3.04 | 10.50±2.17 |
| E | 402.48±198.38 | 148.97±83.60 | 50.92±13.12 | 20.51±5.68 | 12.29±2.18 |
| F | 285.30±193.96 | 207.32±121.08 | 71.01±32.73^¶,‡,†^ | 36.65±14.46^∆,¶,‡,†,§^ | 23.35±6.66^∆,¶,‡,†,§^ |
