# Supplementary material for: α-Ketoglutarate stimulates cell growth through the improvement of glucose and glutamine metabolism in C2C12 cell culture
Source: Front Nutr. 2023 May 10;10:1145236. doi: 10.3389/fnut.2023.1145236 (PMC10208397; doi:10.3389/fnut.2023.1145236)
Supplement: Supplementary file 6 [file Table_6.DOCX]

| Group | Baseline | Day 1 | Day 2 | Day 3 | Day 4 | Day 5 | Day6 | Day7 | Day8 |
| --- | --- | --- | --- | --- | --- | --- | --- | --- | --- |
| A | 1.70±0.24 | 2.57±0.22 | 5.05±0.59 | 11.00±1.63 | 19.26±4.20 | 27.58±4.97 | 29.90±5.31 | 35.33±7.03 | 39.04±3.07 |
| B | 1.83±0.25 | 2.83±0.40 | 5.18±1.36 | 12.42±2.28 | 19.70±4.30 | 29.04±3.62 | 28.06±4.54 | 39.45±2.40 | 38.11±4.25 |
| C | 1.80±0.24 | 2.90±0.30 | 5.50±1.01 | 11.16±1.62 | 19.05±3.68 | 25.51±4.01 | 28.72±1.32 | 34.45±1.99 | 36.35±2.60 |
| D | 2.01±0.30 | 2.80±0.27 | 5.32±0.72 | 11.39±1.97 | 19.23±2.48 | 24.47±3.43 | 26.02±2.38 | 31.03±2.83^¶^ | 32.08±5.32^∆,¶,‡^ |
| E | 1.82±0.35 | 2.76±0.34 | 5.64±0.43 | 11.45±0.81 | 15.29±0.79^¶^ | 17.42±2.38^∆,¶,‡,†^ | 18.05±2.04^∆,¶,‡,†^ | 22.08±3.74^∆,¶,‡,†^ | 24.86±1.25^∆,¶,‡,†^ |
| F | 1.74±0.23 | 2.62±0.26 | 5.03±0.85 | 9.31±1.72^¶^ | 12.65±1.01^∆,¶,‡,†^ | 16.35±1.79^∆,¶,‡,†^ | 17.56±2.18^∆,¶,‡,†^ | 21.62±0.93^∆,¶,‡,†^ | 21.94±0.94^∆,¶,‡,†^ |
