# Supplementary material for: α-Ketoglutarate stimulates cell growth through the improvement of glucose and glutamine metabolism in C2C12 cell culture
Source: Front Nutr. 2023 May 10;10:1145236. doi: 10.3389/fnut.2023.1145236 (PMC10208397; doi:10.3389/fnut.2023.1145236)
Supplement: Supplementary file 7 [file Table_7.DOCX]

| Group | Day 1 | Day 2 | Day 3 | Day 4 | Day 5 |
| --- | --- | --- | --- | --- | --- |
| A | 238.63±196.18 | 1082.09±300.29 | 576.79±115.85 | 422.65±73.62 | 386.57±71.49 |
| B | 368.01±359.95 | 878.70±366.34 | 421.53±58.48^∆^ | 288.06±36.32^∆^ | 256.88±34.21^∆^ |
| C | 386.82±307.99 | 998.18±121.61 | 419.68±60.67^∆^ | 285.10±46.63^∆^ | 268.63±48.94^∆^ |
| D | 269.81±261.24 | 1049.42±546.15 | 510.81±78.53 | 330.57±48.65^∆^ | 309.46±65.09 |
| E | 804.02±127.58^∆,¶,‡,†^ | 1473.25±367.02^¶^ | 590.12±154.78^¶,‡^ | 299.11±47.64^∆^ | 245.71±38.35^∆^ |
| F | 681.08±343.49^∆,¶,†^ | 1358.51±575.40 | 614.79±106.06^¶,‡^ | 416.45±104.16^¶,‡,†,§^ | 344.33±113.37^§^ |
