# Supplementary material for: α-Ketoglutarate stimulates cell growth through the improvement of glucose and glutamine metabolism in C2C12 cell culture
Source: Front Nutr. 2023 May 10;10:1145236. doi: 10.3389/fnut.2023.1145236 (PMC10208397; doi:10.3389/fnut.2023.1145236)
Supplement: Supplementary file 8 [file Table_8.DOCX]

| Group | Baseline | Day 1 | Day 2 | Day 3 | Day 4 | Day 5 | Day6 | Day7 | Day8 |
| --- | --- | --- | --- | --- | --- | --- | --- | --- | --- |
| A | 1.07±0.04 | 1.31±0.03 | 1.47±0.08 | 1.68±0.10 | 1.82±0.11 | 2.09±0.16 | 2.30±0.43 | 2.32±0.46 | 2.34±0.23 |
| B | 1.08±0.08 | 1.28±0.05 | 1.42±0.03 | 1.60±0.07 | 1.91±0.11 | 2.12±0.13 | 1.91±0.36 | 2.26±0.16 | 2.34±0.20 |
| C | 1.03±0.06 | 1.28±0.09 | 1.50±0.06 | 1.62±0.05 | 1.81±0.12 | 2.01±0.16 | 1.98±0.32 | 2.03±0.10 | 2.10±0.10 |
| D | 1.10±0.05 | 1.29±0.07 | 1.49±0.11 | 1.75±0.21 | 2.03±0.32^∆,‡^ | 2.28±0.28^‡^ | 1.88±0.25^∆^ | 2.02±0.18 | 1.99±0.24^∆,¶^ |
| E | 1.09±0.03 | 1.30±0.05 | 1.47±0.07 | 1.69±0.10 | 1.87±0.11 | 2.19±0.17 | 2.08±0.26 | 1.91±0.20^∆,¶^ | 1.91±0.31^∆,¶^ |
| F | 1.03±0.09 | 1.23±0.05 | 1.40±0.09 | 1.57±0.08^†^ | 1.70±0.09^†^ | 1.89±0.12^†,§^ | 1.91±0.22 | 1.96±0.12^∆^ | 1.90±0.15^∆,¶^ |
