# Supplementary material for: α-Ketoglutarate stimulates cell growth through the improvement of glucose and glutamine metabolism in C2C12 cell culture
Source: Front Nutr. 2023 May 10;10:1145236. doi: 10.3389/fnut.2023.1145236 (PMC10208397; doi:10.3389/fnut.2023.1145236)
Supplement: Supplementary file 9 [file Table_9.DOCX]

| Group | Day 1 | Day 2 | Day 3 | Day 4 | Day 5 |
| --- | --- | --- | --- | --- | --- |
| A | 174.41±8.61 | 116.17±9.14 | 28.85±1.23 | 14.36±1.56 | 9.34±3.04 |
| B | 128.09±28.72^∆^ | 68.06±11.85^∆^ | 18.78±2.68^∆^ | 9.61±1.61 | 7.15±1.72 |
| C | 147.53±33.22^∆^ | 86.86±16.62 | 20.01±3.45^∆^ | 9.02±1.90 | 6.17±1.86 |
| D | 122.85±28.98^∆^ | 99.29±46.61 | 27.59±5.69^¶,‡^ | 14.85±4.03^¶,‡^ | 11.83±4.00^¶,‡^ |
| E | 132.23±46.96^∆^ | 81.39±25.77^∆^ | 34.02±8.25^¶,‡^ | 18.19±4.03^∆,¶,‡^ | 15.65±4.74^∆,¶,‡^ |
| F | 124.04±18.75^∆^ | 96.44±13.90 | 38.21±6.55^∆,¶,‡,†^ | 20.66±5.16^∆,¶,‡,†^ | 14.18±4.52^∆,¶,‡^ |
